# Barriers in Access to Dental Services Hindering the Treatment of People with Disabilities: A Systematic Review

**DOI:** 10.1155/2020/9074618

**Published:** 2020-07-23

**Authors:** Saulo V. da Rosa, Samuel J. Moysés, Laís C. Theis, Renata C. Soares, Simone T. Moysés, Renata I. Werneck, Juliana S. Rocha

**Affiliations:** ^1^School of Life Sciences, Pontifical Catholic University of Paraná, Curitiba 80215-901, Paraná, Brazil; ^2^School of Medicine, Pontifical Catholic University of Paraná, Curitiba 80215-901, Paraná, Brazil

## Abstract

**Background:**

People with disabilities tend to have greater oral health problems compared to those without disabilities. This may be due to barriers they come across in accessing dental services.

**Objectives:**

The objective of this systematic review was to provide a critical digest of the scientific literature concerning barriers and facilitators of access to oral health services for people with disabilities.

**Methods:**

The electronic databases PubMed, Scopus, Web of Science, Latin American and Caribbean Health Sciences Literature (LILACS), and Brazilian Library of Dentistry (BBO) were searched using keywords relevant to the subject. The search was not restricted to specific languages or years of publication; all relevant studies were translated and reviewed.

**Results:**

Sixteen studies including 14 articles, a doctoral thesis, and a monograph were selected, and their quality was analysed using the Downs and Black assessment tool. Barriers to dental services were divided into physical or nonphysical based on the dentist's perspective, as per the perception of parents/guardians or by the persons with disabilities. The barriers that emerged included the dentist's lack of preparation to assist people with disabilities, structural problems of access to dental offices, communication difficulties, and lack of awareness regarding the need for dental treatment for the disabled person.

**Conclusion:**

It is concluded that people with disabilities continue to run into complex physical, behavioural, or multidimensional barriers in accessing dental services. Improved training of dentists for the care of this population is hereby emphasized. The legal framework enabling access to dental care for people with disabilities must also be respected in each country.

## 1. Introduction

The number of persons with disabilities worldwide is almost one billion [1]. The affected population is at a higher risk of caries and periodontal disease [2–5] compared to those without disabilities. Epidemiological profiles in oral health show a difference, for example, in the DMFT (decayed, missing, and filled teeth) of people with disabilities when compared to people without disabilities. These differences may vary according to the country and the types of disabilities included in the survey. In South Korea, researchers evaluated comparable samples of people (a) with physical disabilities (DMFT = 7.3), (b) with mental disabilities (DMFT = 8.3), and (c) with multiple disabilities (DMFT = 8.2) and people without disabilities (DMFT = 4.9) [6]. In another study carried out in Portugal, with institutionalized people with disabilities, a mean DMFT of 11.2 was found which was much higher than the general population of the same age group [7]. In Brazil, in a study carried out with people with Down syndrome, cerebral palsy, and intellectual deficit, the average DMFT was 11.0, also quite high when compared to the general population of the same age group [8].

This is partly due to the difficulties encountered in the care of the oral cavity, which include structural barriers and motor difficulties and those associated with communication with the caregiver regarding the need for oral health care [4], in addition to the degree of dependence on caregivers for hygiene and good eating practices [3]. This dependence observed in persons with disabilities can often make their oral care take a back seat for parents or caregivers, as their overall health becomes a priority [9, 10].

When individuals with disabilities take initiative to access health services and gain access, they subsequently choose to continue care in services that have empathetic, compassionate, and responsible professionals [11]. Yet, access to care is deemed to be the first barrier to the initiation of a health service, wherein the patient's problem should be known and their therapeutic itinerary in the service network be traced so as to solve the health problem [12]. Access to health services by persons with disabilities should consider (but not exhaust themselves with) their most basic and specific health needs [5, 13, 14].

Knowledge of the main barriers that hinder oral health care in people with disabilities needs to be discussed. Access to care is determined by the geographical location of the patients and services, the convenience and ability to organize services in order to accommodate the individual, their adaptability to the service provided, and the reciprocal acceptability of professionals and end users [15]. Access is determined by the type of health system in each country and the local context. In order to enable access to health care, the health service must meet the needs of the user, be available, and be appropriate to the population.

Thus, universal access to health services comprise (i) a political aspect involving decision-making and agreement by the different levels of the government in a country, thereby prioritizing and programmatically arranging the services and the intervention devices in context; (ii) economic and social aspects that cover financing issues and mitigation or elimination of barriers encountered by the user; (iii) system organization starting from the user's entry into the service up till the resolution of their health problem; and (iv) technical-scientific training and cultural competence, through comprehensive actions and respect for individuals, understanding the health-disease transitions, beliefs, values, and traditions [16].

Based on the above, the objective of this systematic review was to critically gather from the scientific literature the main barriers and facilitators of access to oral health services for people with disabilities.

## 2. Materials and Methods

### 2.1. Search Strategy

This systematic review was carried out according to the Cochrane Collaboration Guidelines Combined [17, 18], Meta-analyses Of Observational Studies in Epidemiology (MOOSE) [19], and according to the model Preferred Reporting Items for Systematic reviews and Meta-Analysis guide (PRISMA) [20]. Prior to its initiation, the protocol for this review was registered at the PROSPERO database (protocol number: CRD42018107571).

The guiding question of the review was defined as follows: How do people with disabilities access oral health services?

The keywords were selected based on the study question and study population.

Online databases were consulted from August 24, 2018, to August 27, 2018, based on the date of online availability and included PubMed, Scopus, Web of Science, Latin American and Caribbean Health Sciences Literature (LILACS), and the Brazilian Library of Dentistry (BBO). In addition, the references cited in all the primary studies included were manually searched to add all the relevant publications that may not have been included in the main search. At this stage, the grey literature was also accessed through the ProQuest Dissertations and Theses Full-Text databases, CAPES Theses Periodicals, the Grey Literature Report, and Google Scholar. Abstracts from the annual conference of the International Association for Dental Research (IADR) and its regional divisions (1990–2018) were also searched.

The search strategy was appropriately modified for each database; two reviewers (SVR and LCT) performed the search in order to identify eligible studies.  Table 1 depicts the details of the search conducted with the search date and number of articles found in each database.

### 2.2. Eligibility Criteria

Though observational studies (cross-sectional, cohort, and case-control) were included, editorial letters, historical reviews, in vitro studies, controlled trials, case reports, comments, and qualitative studies were excluded from the present study. No language or publication date restrictions were imposed; all relevant studies were translated and revised.

### 2.3. Data Collection and Analysis Process

**Figure 1:**
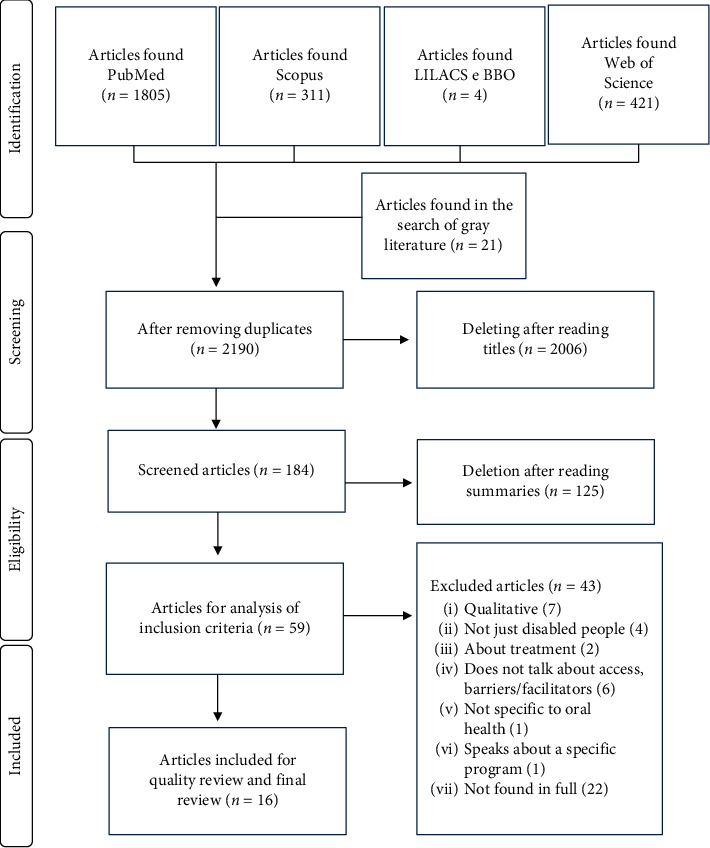
Flowchart of the study selection process according to the PRISMA guidelines.

After the exclusion of duplicates, using the ®Mendeley reference manager, studies were initially screened on the basis of titles and abstracts. Articles that appeared in more than one database were considered only once. The full-text versions of the articles were evaluated by a pair of reviewers (SVR and LCT) who determined study eligibility; disagreements were decided by a third reviewer (JSR). Each eligible article was assigned an identification code (first author/year) to facilitate its classification. Inter-rater reliability was calculated by Kappa statistic; a value of 0.87 was considered optimal. Relevant information on study design, participant characteristics, exposure, and outcomes was extracted through a customized pretested data extraction form (Figure 1).

### 2.4. Bias Risk Assessment

The quality of the articles was assessed by two independent reviewers (SVR and LCT) using the Downs and Black scale [21]. This instrument is used for quality assessment of observational studies and randomized clinical studies and comprises 27 items totalling up to 32 points (higher scores indicating superior quality). In this review, a modified version [22–28] of this instrument was used which consisted of 17 items (1–3, 5–7, 9–11, 16–18, 20–22, and 25–26), totalling up to a maximum of 17 points. The relevant domains for the instrument included description, external validity, and internal validity (confusion/selection bias). Disagreements in the quality of the articles were resolved by a third reviewer (JSR).

For each aspect of quality assessment, the risk of bias was scored according to an adapted version of the Cochrane Collaboration tool [17], which included the top four domains from the Downs and Black quality assessment tool. Studies were considered to have a “low” risk of bias when the domains external validity, internal validity, and description attained their maximum scores. A single study presented a risk of “unclear” bias when the criterion description was not fulfilled and/or was unclear in the other key domains. A “high” risk of bias was considered when a study did not meet the criteria internal and external validity, and when more than two items reflected a high risk of bias in the domain description.

Kappa was also performed to measure inter-rater agreement and to analyse the risk of raters' classification bias. A Kappa value of 0.86 was attained, which is considered optimal as per the relevant literature.

## 3. Results

### 3.1. Summary of Results

Data were analysed using the extracted information which included the study title, author, year, country, sample size, study design, study results (barriers encountered in dental care, types of services accessed, type of dental procedures, and types of disabilities), and source of the study population. Because of the heterogeneity of the studies, a meta-analysis was not performed. The target audience of the questionnaires were people with disabilities; however, the dentist was interviewed. Also, the diversity among the studies was due to the age or the age groups of the target population, the difference in the health systems in the countries studied, and differences in the type of disability.

### 3.2. Characteristics of Included Studies

A total of 2,190 articles were derived from all the databases after removal of duplicates (Figure 1). However, the number reduced to 59 articles following careful reading of titles and summaries. Subsequent to reading the full-text versions, 43 articles were excluded for the reasons such as (i) use of a qualitative approach (*n* = 7) [29–35]; (ii) included subjects other than people with disabilities (*n* = 4) [36–39]; (iii) did not discuss access, barriers, or facilitators of care (*n* = 6) [40–45]; (iv) did not specifically address oral health (*n* = 1) [46]; (v) were highly restricted and dealt with only a specific program (*n* = 1) [47]; and (vi) were not available in full text for reading (*n* = 22) [48–69].

Table 2 depicts a summary of the characteristics of the 16 studies that were finally included. It is noteworthy that one was a doctoral thesis and another a monograph (grey literature). The studies originated in several countries and continents; six studies were from North America with four from the United States [70, 72, 74, 77] and two from Canada [71, 76]. There were six studies from Brazil in South America [5, 79, 80, 82–84]. Two selected studies were from Europe, of which one was from England [73] and the other from the Netherlands [75]. A single article was selected from Malaysia [81] and another from Australia [78].

Only two studies were derived from a secondary database [72, 74], and the other fourteen discussed data from primary sources that had a cross-sectional observational study design.

### 3.3. Bias Risk Assessment

The results of the bias risk assessment are shown in Table 3. One study showed a low risk of bias for all the assessed items [74]. Though, three studies depicted an unclear risk of bias [70, 72, 84], seven of them showed a high risk of bias due to ambiguity in the description of confounding factors and their adjustment during the selection of the study participants [5, 71, 76, 79–82]. With respect to external validity, three articles presented a high risk of bias [70, 82] and one presented an unclear risk [75]. At this stage, the extent to which the study conclusions could be extrapolated to the studied population was evaluated. On assessing internal validity (systematic error), only one article showed a high risk of bias, the main outcomes of which were not accurate and the data were only descriptively depicted [79]. In addition, on evaluating internal validity in terms of confusion and selection bias, only two studies showed low risk of bias due to control in sample selection, wherein data were collected from the same population and over the same time period [72, 74] and one study showed unclear risk of bias [84].

### 3.4. Summary of the Main Barriers

The barriers observed in this review were classified as physical or nonphysical or classified based on the perception of the person responsible for, or the caregiver of the disabled person involved in the study, and the perception of the dental surgeon attending to the disabled person. The results of the barriers detected are reported in Table 4.

Common barriers observed among the included articles comprised the cost of treatment [71, 72, 76, 82, 83], the dentist's lack of preparation for dental care of the disabled persons [5, 70, 75–78, 80, 81, 84], inadequacy of dental facilities that were accessible to the disabled [70, 76, 80, 84], and lack of adaptation of the access routes to the health care facilities and dental offices [76, 77, 79, 81]. None of the selected studies discussed facilitators of access to oral health services for people with disabilities.

Table 2 shows the types of services used by people with disabilities as reported by their caregivers or by the dental surgeon interviewed. In the United States, the services availed were those remunerated by health plans and federal government funds [70, 72, 74, 77]. In Malaysia, the mainly availed services were private [81]; in Canada, there were private services and those paid by social, federal, and provincial institutions [71, 76]. Services were offered by private, public, and social institutions in Brazil [5, 79, 80, 82–84]. Eleven of the 16 studies reported the services available [70, 71, 74, 76, 79–84].

The types of disabilities addressed in the studies were rather broad and are shown in Table 2. These included physical, mental, hearing, or visual disabilities and syndromes. Most common disabilities in studies that appeared more than once and are associated with selected conditions were also specified and included conditions such as autism [71, 72, 76–78, 84], cerebral palsy [71–74, 76–78, 80, 82, 84], mental retardation [71, 72], developmental delay [72, 76, 77], cleft palate [74, 77], spina bifida [74, 78], Down's syndrome [72, 76, 77, 80, 82, 84], intellectual disability [75, 78, 81, 82, 84], Rett syndrome [80, 84], motor disability [5, 84], and hearing deficiency [5, 79, 84].

The dental procedures reported in the aforementioned studies also highlighted the treatment needs of the population. In people with disabilities, the simplest procedures can be difficult to perform due to problems associated with communication or physical constraints such as muscle stiffness, poor mouth opening, and resistance to treatment that often led to care under general anesthesia [85]. Four of the selected studies reported emergency treatment procedures [70, 74, 77, 81]; dental extractions and other types of surgeries [71, 76, 78, 80–83] and preventive procedures such as prophylaxis, sealants, and fluoride application were also reported [70, 71, 76–78, 80, 82, 83].

## 4. Discussion

As this population needs specially organized health services and comprehensive preparation of professionals [85], it was observed that barriers to access were clearly pointed and appeared in all the included articles. However, there was no mention of the facilitators of access in any of the studies.

The design of all included studies showed a cross-sectional framework allowing to simply estimate the prevalence of evaluated variables or at most their relationship. On the other hand, in addition to cross-sectional studies not being adequate to analyse causality, just association, another problem is the possibility of a low response rate of participants. Therefore, the researcher needs to make use of sample contact strategies such as telephone and mail communication. In the results of the studies included in this systematic review, the response rate of the questionnaires sent was reported in ten studies, not mentioning significant sample losses, suggesting that the response rate did not affect the results. For the others, for not having addressed this issue, it is unknown [70, 71, 74–78, 81, 83, 86]. It is emphasized that the sample selected for the study must be representative of the entire population studied, so that the results can be extrapolated. In addition to the risk of obtaining low responses, there is also a likelihood of biased responses [87].

Studies that were not found in their entirety and excluded from the final selection in this systematic review were mostly those reported during the period from the 1950s up to the 1980s. Not including these studies in the analysis of the results hampered the possibility of revealing old and persistent barriers to dental services. This resulted in compromising the analysis due to a possible change in the nature of barriers over a longer time interval [48–69].

In the analysis of the study quality, the “report” item assessed whether the information provided by the study was sufficient for the reader to make an unbiased assessment of the conclusions derived from the study [21]. Examining this dimension, in this review, showed that six studies had a low risk of bias thereby implying that they addressed the requirements of the Downs and Black instrument which allows the reader to make an unbiased assessment [74, 76–78, 83, 86]. In contrast, seven studies did not meet these requirements [5, 71, 79–82, 84]. External validity determines the extent to which the study results can be extrapolated to the population studied; seven of the 16 studies had a low risk of bias [72–78, 83], being reliable results for the sample of the studied population. With regard to internal validity (confusion and selection bias) only two studies reported a low risk of bias [72, 74], thereby implying that the biases related to sample selection were addressed and the interventions quantified in the results. In this regard, thirteen studies showed high risk of bias [5, 70, 71, 75–82, 84]. These studies presented only the descriptive results pertaining to the data in the form of frequency of answers and percentages and did not perform statistical adjustments of the results.

Physical barriers reflect problems relating to accessibility that people with disabilities encounter to reach a dental care facility. Two articles reported physical barriers hindering access to care facilities, such as surgeries being performed on the upper floors of buildings that did not have elevators and not remembering that dental extraction is a common requirement among people with disabilities [75, 81]. Inadequate dental facilities are also barriers that affect access [70, 76, 80, 84]. The compromised mobility of people with disabilities affecting their ability to reach the place of care is yet another critical barrier and has been reported in three studies included in this review [76, 82, 83]. Difficulties in access due to lack of adaptation to health care facilities and offices has been mentioned in four studies [76, 77, 79, 81]. To overcome such barriers, dental offices and dental centres must follow and abide by accessibility laws as enforced in many countries such as Brazil, as access to dental care is a right of the disabled.

Family involvement in access to care for people with disabilities is very essential, as family support and emotional bond play a fundamental role in their health. Expecting a child with disabilities can inflict emotional distress and guilt in parents, who are required to prepare appropriately and introject situations of difficulty that they may encounter, given the social, structural, and programmatic inequities that place a disabled person in a vulnerable situation [88]. The family of children with disabilities may be in denial upon identification of the condition, as they lack preparedness to care for a child with a disability. They tend to adapt as they seek information in order to meet the care needs of their loved ones. Subsequently, they enter a phase of acceptance, when they establish an emotional bond with their child and understand their health care needs [89].

People with disabilities often have a number of associated health problems such that oral health care takes a back seat in the family [10]. This barrier to oral health care was also detected by Nelson et al. [77]. Koneru and Sigal [76] also encountered a perceived lack of dental treatment in parents or caregivers. The dearth of time to take the affected child or the guardian to the dentist was reported by Nelson et al. [77] and Dantas Cardoso [82]. Cardoso et al. [80] cited instances in which as per the perception of the parents or caregivers, the child with disability did not need care. Instances depicted by Nelson et al. [77] reflect the lack of knowledge regarding the need for oral health care. These included situations where the child was deemed to be too young to see a dentist, the father feared a visit to the dentist, or when the child had recently exfoliated his or her deciduous teeth. Therefore, access to information and health education for parents and caregivers of people with disabilities can overcome these barriers [90].

The studies selected belonged to various parts of the world, with representation from the United States [70, 72, 74, 77], Canada [71, 76], Netherlands [73], Malaysia [81], Australia [78], England [73], and Brazil [5, 79, 80, 82–84]. Although these countries have diverse health systems, barriers to care reported in these studies were similar. One of the most common barriers was the lack of preparation and experience of the professional for dental care of disabled persons [5, 70, 75, 77–80, 84]. Knowledge is fundamental for good dental practice, and innovation and the use of instruments that can facilitate the time of care can always help in the treatment of people with disabilities when we do not have a collaboration during the consultation [91]. This is a significant finding, which reflects upon the training received by the dentist or even the dental curriculum, which does not consistently cover the theme of dental care for people with disabilities [92]. In such situations, continuing health education programs can aid dental professionals to keep abreast of the techniques to meet the needs of people with disabilities.

Permanent health education allows professionals to refresh their knowledge and practices through latest evidence and the best treatment approach available, thereby enabling the enhancement of technical skills, scientific knowledge, and ethical development of the processes and also building relationships between the teams involved. The appropriate distribution of professionals and services to the proximity of the population in need, irrespective of their location with an aim to improve access to care and to enable continuing health education, is a complex task, as there is a noticeable concentration of specialized professionals in large centres [93]. This imposes alternatives such as the virtualization of learning through nonpresent or semipresent modules.

Most of the studies included in the review were centred primarily in countries such as Brazil, the United States, and Canada. Brazil has a universal Unified Health System (SUS) enshrined in its federal constitution of 1988. It is a free system accessible to all Brazilian citizens, including people with disabilities. It has doctrinal principles: universality, equity, and comprehensiveness [94, 95]. Primary care is the gateway to the SUS; there are family health teams (FHS) which aim at comprehensive multidisciplinary care of the people [96]. In the year 2000, dentists were included in this team with the objective of oral health actions and services as a part of primary care [96]. Among the barriers observed in the studies conducted in Brazil, the lack of trained professionals for the dental care of people with disabilities was prominent, given that these surveys were conducted among people who attended the SUS [5, 79, 80, 82–84].

In the United States, the most frequent barrier was the lack of experience among dentists and the cost of treatment [70, 72, 74, 77]. The latter reflected the characteristic of the country's health system, whereby there was no universal system followed, and one had to pay for health insurance or use Medicare and Medicaid, which are subsidized by the government for vulnerable groups of patients [97]. In Canada, the health system is provincial with variations in each province; however, access to oral health services is mostly not covered by this system [98]. The type of health system in a country is therefore a crucial determinant of the physical barriers and the cost of treatment [71, 76].

The difficulty to provide dental care and the lack of trained professionals to care for people with disabilities has a direct influence on their oral health. The procedures that need to be performed are most often on patients requiring urgent care where they are already in pain, mandating tooth extraction due to decay or prophylaxis due to bacterial biofilm accumulation. Procedures such as restorations and preventive treatments can be performed in order to prevent tooth mutilation. The use of prostheses can also restore the well-being and quality of life of people with disabilities [70, 71, 74, 76–78, 80–83].

The compilation of the barriers observed was derived from studies using quantitative methods which in turn may be a limitation of this systematic review, considering that qualitative studies may approach barriers differently. For example, qualitative studies are ideally not limited to structured questions with closed options, and one's view of access to services can be further explored, thus opening horizons for another systematic review (metasynthesis) for qualitative studies. Another limitation to be considered was the inclusion of only cross-sectional studies. Finally, it is alarming that facilitators of access to dental services for people with disabilities have not been discussed or reported in these revised studies, which paves way for further studies in this field, aiming at solving the barriers that hinder access to dental services.

## 5. Conclusion

People with disabilities continue to encounter various physical, structural, geographical, professional, or behavioural barriers that hinder their access to dental services. Furthermore, there is a need to improve the training rendered to dentists pertaining to care for this population in various national and regional contexts. It would be ideal to enforce and implement accessibility laws in every country. Therefore, a lot remains to be achieved by the society with regard to the facilitation of access to health care. Overcoming the barriers encountered by people with disabilities can thereby enable their much deserved and dignified access to oral health services.

## Figures and Tables

**Table 1:** Search strategy in electronic databases (August 24–August 27, 2018).

PubMed—August 24, 2018	1805 results

# 1 ((((((((((((((((((((((dental health Services [MeSH terms] OR “dental health services” [title/Abstract]) OR “health services”[Title/Abstract]) OR “health services Accessibility”[Title/Abstract]) OR “dental Care”[Title/Abstract]) OR “dental care for Disabled”[Title/Abstract]) OR “health services for persons with Disabilities”[Title/Abstract]) OR “utilization of health services”[Title/Abstract]) OR “health services utilization”[Title/Abstract]))	#2 (((((((((((disabled persons [MeSH terms) OR “disabled persons”[Title/Abstract]) OR “disabled person”[Title/Abstract]) OR “persons with Disabilities”[Title/Abstract]) OR “persons with Disability”[Title/Abstract])

#1AND#2	

LILACS e BBO—August 27, 2018	4 results

# 1 (mh:(“Dental health services”)) OR (tw:(“Dental health services”)) OR (tw:(“Serviços de saúde bucal”)) OR (tw:(“Servicios de salud dental”)) OR (tw:(“Health services”)) OR (tw:(“Serviços de saúde”)) OR (tw:(“Servicios de salud”)) OR (tw:(“Acesso aos serviços de saúde”)) OR (tw:(“Accesibilidad a los servicios de salud”)) OR (tw:(“Assistência odontológica”)) OR (tw:(“Atención odontológica”)) OR (tw:(“Health services accessibility”)) OR (tw:(“Dental care”)) OR (tw:(“Assistência Odontológica para Pessoas com deficiências”)) OR (tw:(“Atención Dental para Personas con discapacidades”)) OR (tw:(“Dental care for disabled”)) OR (tw:(“Health services for persons with disabilities”)) OR (tw:(“Serviços de Saúde para Pessoas com deficiência”)) OR (tw:(“servicios de Salud para Personas con discapacidad”))	#2 (tw:(“Disabled persons”)) OR (tw:(“Pessoas com deficiência”)) OR (tw:(“Personas con discapacidad”)) OR (mh:(“Disabled persons”)) OR (mh:(“Disabled person”)) OR (tw:(“Disabled person”)) OR (tw:(“Pessoa com deficiência”)) OR (tw:(“Persona con discapacidad”))

#1AND#2	

Web of Science—August 27, 2018	421 results

# 1 Tópico: (“Dental health services”) *OR* TÓPICO: (“Health services”) *OR* TÓPICO: (“Health services accessibility”) *OR*TÓPICO: (“Persons with disability”) *OR* TÓPICO: (“Dental care”) *OR* TÓPICO: (“Dental care for disabled”) *OR*TÓPICO: (“Health services for persons with disabilities”) *OR* TÓPICO: (“Utilization of health services”)	#2 (“disabled persons”) *OR* TÓPICO: (“Persons with Disabilities”) *OR* TÓPICO: (“Disabled person”) *OR*TÓPICO: (“Persons with disability”)

*Índices* *=* *SCI-EXPANDED, SSCI, A&HCI, CPCI-S, CPCI-SSH, ESCI Tempo estipulado* *=* *Todos os anos*	*Índices* *=* *SCI-EXPANDED, SSCI, A&HCI, CPCI-S, CPCI-SSH, ESCI Tempo estipulado* *=* *Todos os anos*

#1AND#2	

Scopus—August 27, 2018	311 results

# 1 TITLE-ABS-KEY (“disabled persons”) OR TITLE-ABS-KEY (“disabled person”) OR TITLE-ABS-KEY (“persons with disabilities”) OR TITLE-ABS-KEY (“persons with disability”)) AND (LIMIT-TO (SUBJAREA, “DENT”))	#2 (TITLE-ABS-KEY (“Dental health Services”) OR TITLE-ABS-KEY (“Health services”) OR TITLE-ABS-KEY (“Health services Accessibility”) OR TITLE-ABS-KEY (“Dental Care”) OR TITLE-ABS-KEY (“Dental care for Disabled”) OR TITLE-ABS-KEY (“Health services for persons with Disabilities”) OR TITLE-ABS-KEY (“Utilization of health services”) OR TITLE-ABS-KEY (“Health services utilization”)

#1AND#2	

**Table 2:** Characteristics of the studies.

Author/year	Country	Study design	Type	Sample	Sample features	Statistical analysis	Types of services	Types of disabilities	Most frequent dental procedures
Burtner et al, 1990 [70]	United States	Cross-sectional	Article	362 respondents	Persons with disabilities from the Florida Department of Health and Rehabilitation, United States. Primary data.	Not adjusted	Treatment paid by federal government funds. Private payment through private plans or funds.	—	Emergency treatment, examination and prophylaxis, other services.

Milnes et al, 1995 [71]	Canada	Cross-sectional	Article	342 respondents	Manitoba dentists. Primary data.	Not adjusted	Private, social, federal, provincial, municipal, unpaid services, institutions.	Asthma, autism, cancer, cardiac arrest, cerebral palsy, diabetes, hearing impaired, hemophilia, immunosuppression, leukemia, liver, mental retardation, renal, spinal cord injury, stroke, visually impaired.	Examinations, oral radiographs, panoramic radiography, topical fluoride, restoration, scaling, crown and bridge, partial and total prosthesis, periodontics, endodontics, surgery, orthodontics and sealants.

Schultz et al, 2001 [72]	United States	Cross-sectional	Article	12,539	The data source for this study was an interview survey of the National Center for Health Statistics 1997. Secondary data.	Adjusted	—	Mental retardation, cerebral palsy, attention deficit, Down syndrome, autism, developmental delay.	—

Edwards et al, 2002 [73]	England	Cross-sectional	Article	157 respondents	Liverpool dentists, Sefton and St Helens and Knowsley. Primary data.	Not adjusted	—	Physical disability, learning disabilities, and mental health problems.	—

Al Agili et al, 2004 [74]	United States	Cross-sectional	Article	714 respondents	Database provided by child rehabilitation services from Alabama. Secondary data.	Adjusted	Private payment through private plans or funds.	Cleft lip and/or palate or other craniofacial disorders; epileptic/convulsive disorders; spina bifida; cerebral palsy.	Seek emergency treatment rather than prevention due to barriers encountered.

De Jongh et al, 2008 [75]	Netherlands	Cross-sectional	Article	40 dentists and 126 responding caregivers.	Children with disabilities in day care centres in the Netherlands. Primary data.	Not adjusted	—	Most subjects suffered from physical disabilities and complex medical problems. Based on their social aspects, language, and motor skills, all children were considered to have a severe mental disability.	—

Koneru and Sigal, 2009 [76]	Canada	Cross-sectional	Article	634 respondents	People with disabilities in Ontario.	Not adjusted	Private insurance, Ontario Disability Support Program, another form of government-sponsored coverage.	Autism, cerebral palsy, Down syndrome, developmental delay, physical disability, psychiatric disability, brain injury.	Examination, X-ray, fluoride application, cleaning, oral hygiene instruction, sealants, fillings, extraction, stainless steel crown, aesthetic crown and bridge, dentures, whitening, canal treatment, dental implants.

Nelson et al, 2011 [77]	United States	Cross-sectional	Article	1128 respondents	Children with disabilities in Massachusetts, United States.	Not adjusted	—	Autism, invasive developmental disorder, Asperger's syndrome, cerebral palsy, musculoskeletal disorders, seizures, cystic fibrosis, developmental/neurological/behavioral/chromosomal delay, Down syndrome, speech/hearing/blind, metabolic/cardiac/renal/immune, hemophilia/sickle cell anemia/von Willebrand's disease, craniofacial/cleft lip and palate.	Checkup or just cleaning, emergency services and catering.

Pradhan et al, 2009 [78]	Australia	Cross-sectional	Article	1280 respondents	Adults with disabilities living in Adelaide, South Australia. Primary data.	Adjusted	—	Autism, brain injury, cerebral palsy, intellectual disability, spina bifida and quadriplegia.	Checkup, extractions, restorations, prostheses, and radiographs.

Aragão et al, 2011 [79]	Brazil	Cross-sectional	Article	113 respondents	Disabled children from Recife, Pernambuco, Brazil. Primary data.	Not adjusted	Public service	Physical, mental, hearing	—

Cardoso et al, 2011 [80]	Brazil	Cross-sectional	Article	43 respondents	Caregivers of children with motor disabilities in the city of João Pessoa, Paraíba, Brazil. Primary data.	Not adjusted	Private and public service	Cerebral palsy, hydrocephalus, myelomeningocele, Rett syndrome, Down syndrome, West syndrome.	Prevention, prophylaxis, restoration, extraction, scraping, trauma, orthodontics.

Rocha et al, 2015 [5]	Brazil	Cross-sectional	Article	89 dentists and 204 people with disabilities	Dentists and people with disabilities in Fortaleza, Ceará, Brazil. Primary data.	Not adjusted	Private and public service	Motor impairment, hearing impairment, and visual impairment.	—

Bindal et al, 2015 [81]	Malaysia	Cross-sectional	Article	102 respondents	Dentists from the cities of Kuala Lumpur, Penang, and Kuching in Malaysia. Primary data.	Not adjusted	Private	Physical disability, mental disability, sensory deficiency.	Emergency, extractions, restorative treatment, prostheses, periodontal treatment.

Dantas Cardoso 2015 [82]	Brazil	Cross-sectional	Completion of course work	100 respondents	Parents or guardians of institutionalized patients in APAE/natal. Primary data.	Not adjusted	The most suitable place of care was the Association of Parents and Friends of the Exceptional (APAE)	Mental disability, physical disability, cerebral palsy, birth defect, Down syndrome, sensory impairment, oral communication impairment, behavioral disorder, and autism.	Prophylaxis, restoration, and extraction.

Damiance, 2016 [83]	Brazil	Cross-sectional	Thesis	41 respondents	People with multiple disabilities in the state of São Paulo, Brazil. Primary data.	Adjusted	Philanthropic service, public and private service	Multiple disability.	Review, prevention, checkup, extraction.

Paulo et al, 2017 [84]	Brazil	Cross-sectional	Article	121 respondents	Responsible for people with special needs in João Pessoa, Paraíba, Brazil. Primary data.	Not adjusted	Public service	Mental impairment, cerebral palsy, other motor defects, Down syndrome, Apert syndrome, Rett syndrome, Seckel syndrome, congenital microcephaly, autism, oral communication disorder (speech), audiocommunication disorder (deaf).	—

**Table 3:** Summary of the quality and risk of bias assessment.

Included studies	Risk of bias assessment^*∗*^				Quality assessment^*∗∗*^ (total score)
	Reporting	External validity	Internal Validity - Bias	Internal validity - confusion and selection bias	
Al Agili et al. (2004) [74]	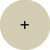	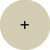	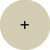	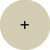	16
Damiance (2016) [83]	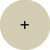	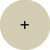	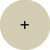	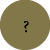	15
Schultz et al. (2001) [72]	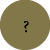	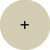	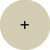	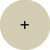	15
Pradhan et al. (2009) [78]	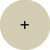	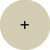	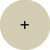	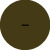	15
Koneru and Sigal (2009) [76]	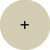	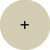	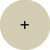	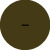	14
Nelson et al. (2011) [77]	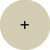	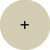	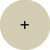	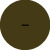	14
Edwards et al. (2002) [73]	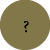	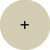	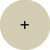	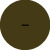	12
Milnes et al. (1995) [71]	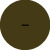	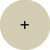	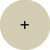	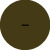	12
De Jongh et al. (2008) [75]	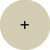	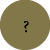	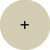	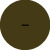	12
Rocha et al. (2015) [5]	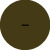	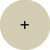	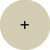	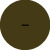	11
Cardoso et al. (2011) [80]	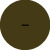	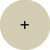	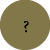	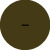	10
Dantas Cardoso et al. (2015) [82]	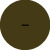	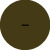	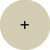	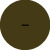	10
Burtner et al. (1990) [70]	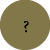	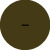	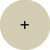	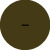	9
Paulo et al. (2017) [84]	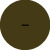	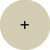	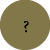	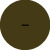	9
Bindal et al. (2015) [81]	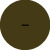	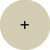	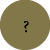	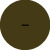	8
Aragão et al. (2011) [79]	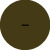	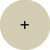	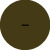	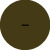	8

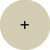
, low risk; 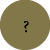, unclear risk; 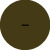, high risk. ^∗^Adapted from Cochrane Collaboration. ^∗∗^Adapted from Downs and Black, scores from zero to 17 (higher scores indicate higher quality).

**Table 4:** Barriers found in the studies.

Author/year	Country	Barriers			
		Physical		Nonphysical	
		Dentist's perception	Caregiver/responsible perception	Dentist's perception	Caregiver/responsible perception
Burtner et al. 1990 [70]	United States	—	(1) The office is not properly equipped.	—	(1) Medicaid or department of health and rehabilitation services does not pay enough (2) The dentist is not trained to deal with patients with disabilities (3) The patient is uncooperative (4) The dentist is too busy with other patients

Milnes et al., 1995 [71]	Canada	—	—	(1) Cost of treatment	—

Schultz et al., 2001 [72]	United States	—	—	(1) Cost of treatment	—

Edwards et al, 2002 [73]	England	(1) Surgery on floors of buildings without elevators. (2) Lack of home care equipment.	—	(1) Lack of time	—

Al Agili et al., 2004 [74]	United States	—	—	(1) Dentist is not willing to treat (2) Health plan not accepted (3) Very young child (4) Not important care (5) Lack of dentist's knowledge to treat	—

De Jongh et al., 2008 [75]	Netherlands	—	—	—	(1) Communication problems (2) Lack of funding (3) Lack of dentist experience in treating children with mental disabilities

Koneru and Sigal, 2009 [76]	Canada	—	(1) Difficulty with physical access (2) Factors of distance (3) Shipping problems (4) Inadequate dental facilities	—	(1) Factors of time (2) Lack of perceived need (3) Fear (4) Cost (5) Inadequate dental training (6) Difficulty communicating pain

Nelson et al., 2011 [77]	United States	—	(1) Difficult to find an affordable dentist's office for the disabled	—	(1) Difficult to take time off from work to bring child to the dentist (2) Difficult to find dentist willing to treat child because of their medical condition (3) Dental care is very expensive (4) Difficult to find a child dentist nearby (5) Difficult to travel to the dental office (6) The dental team is anxious or nervous treating children (7) Child is afraid of the dentist (8) Child does not like to do anything to his mouth (9) The child is too young to see a dentist (10) Dad is afraid to go to the dentis (11) The child has only recently fallen milk teeth (12) The child has other, more urgent health care needs

Pradhan et al., 2009 [78]	Australia	—	—	—	(1) Lack of dentists with adequate skills in managing people with disabilities (2) Cost of treatment (3) Inconvenient location of clinic (4) Lack of dentists willing to treat people with disabilities

Aragão et al., 2011 [79]	Brazil	—	(1) Difficulties in getting to the service due to lack of adaptation of access routes to the health unit for people with walking difficulties (2) Difficulties in service entrance due to lack of adaptation of the building structure	—	(1) Does not have dentist in health unit (2) Fear; the patient refuses to go (3) Difficult to get vacancy (4) Long service (5) Does not like the service

Cardoso et al., 2011 [80]	Brazil	—	(1) Location (2) Lack of structure	—	(1) Low dentist offer for special needs patients (2) Delay in scheduling (3) Unavailability to perform under general anesthesia (4) Do not find the service (5) The child has no need for care (6) The child does not collaborate (7) Lack of humanization of the dentist (8) Time (9) Lack of professional preparation

Rocha et al., 2015 [5]	Brazil	—	—	(1) They do not feel qualified to work with people with special needs due to the difficulty of clinical management of these patients (2) Difficulties communicating with disabled patients, especially with deaf people	(1) Difficulty in receiving dental care (2) Dentists had no special training to work with patients with disabilities

Bindal et al., 2015 [81]	Malásia	(1) Physical barriers to access your clinics (2) There was no ground floor operating room (3) Inaccessible bathrooms (4) Lack of equipment	—	(1) Difficulty in managing patient behavior (2) Communication (3) Time restriction (4) Lack of training (5) Did not have adequate exposure during undergraduate dental studies for special needs	—

Dantas Cardoso, 2015 [82]	Brazil	—	—	—	(1) Lack of vacancies (2) Professional denied attendance (3) Does not have time available (4) Lack of will on the part of the patient (5) Does not have dental services near the residence (6) High shipping cost to carry the patient

Damiance, 2016 [83]	Brazil	—	(1) Transportation	—	(1) Financial

Paulo et al., 2017 [84]	Brazil	—	(1) Location (2) Lack of structure	—	(1) Low dentist offer for special needs patients (2) Delay in scheduling. (3) Unavailability to perform under general anesthesia (4) Do not find the service (5) The child has no need for care (6) The child does not collaborate (7) Lack of humanization of the dentist (8) Time (9) Lack of professional preparation

## Data Availability

The data file of this study is available from the corresponding author upon request.
